# Memories and mimics: unveiling the potential of FDG-PET in guiding therapeutic approaches for neurodegenerative cognitive disorders

**DOI:** 10.3389/fneur.2024.1428036

**Published:** 2024-11-19

**Authors:** Brendan Huang, Sara Sawicki, Carolyn Habiger, Paul J. Mattis, Marc L. Gordon, Ana M. Franceschi, Luca Giliberto

**Affiliations:** ^1^Department of Neurology, Northwell, New Hyde Park, NY, United States; ^2^Zucker School of Medicine at Hofstra/Northwell, Hempstead, NY, United States; ^3^Department of Psychiatry, Northwell, New Hyde Park, NY, United States; ^4^Departments of Neurology and Psychiatry, Donald and Barbara Zucker School of Medicine at Hofstra/Northwell, Hempstead, NY, United States; ^5^Feinstein Institute for Medical Research, Northwell Health, Manhasset, NY, United States; ^6^Department of Radiology, Northwell, New Hyde Park, NY, United States

**Keywords:** Alzheimer’s disease, lewy body dementia, FDG-PET, frontotemporal dementia, systematic review

## Abstract

Fluorodeoxyglucose F18 (FDG) positron emission tomography (PET) imaging can help clinicians pursue the differential diagnosis of various neurodegenerative diseases. It has become an invaluable diagnostic tool in routine clinical practice in conjunction with computed tomography (CT) imaging, magnetic resonance imaging (MRI), and biomarker studies. We present a single-institution case series and systematic literature review, showing how FDG-PET imaging has helped physicians diagnose neurodegenerative diseases and their mimickers and how patient care was amended. A single institution analysis and comprehensive literature search were completed following Preferred Reporting Items for Systematic Reviews and Meta-Analyses (PRISMA) guidelines. These medical subjects’ headings (MeSH) terms were used: “FDG-PET” AND “dementia” OR “Alzheimer’s” OR “neurodegeneration” OR “frontotemporal dementia” OR “atypical parkinsonian syndrome” OR “primary progressive aphasia” OR “lewy body dementia.” The inclusion criteria included studies with uncertain diagnoses of neurocognitive disease resolved with FDG-PET, PET/MRI, or PET/CT hybrid imaging. A literature search resulted in 3,976 articles. After considering inclusion and exclusion criteria, 14 case reports and 1 case series were selected, representing 19 patients. The average age of patients was 70.8 years (range: 54–83 years). Five of the 19 patients were females. Dementia with Lewy bodies (DLB) had the highest propensity for being misidentified as another neurodegenerative disease, followed by Alzheimer’s disease (AD) and frontotemporal dementia (FTD). Without accurate molecular imaging, neurodegenerative diseases may be missed or misdiagnosed. Our single-institution case series and literature review demonstrate how FDG-PET brain imaging can be used to correct and clarify preexisting clinical diagnoses of neurodegenerative disease.

## Introduction

FDG-PET, or [^18^F]-fluorodeoxyglucose positron emission tomography, is a powerful tool in medical research. Since its introduction in Alzheimer’s disease (AD) in the 1980s, FDG-PET has complemented the clinical examination and cerebrospinal fluid (CSF) serology by providing additional information on the pathophysiology of many neurodegenerative diseases (1). With overlapping symptoms between various neurodegenerative diseases, FDG-PET can help to clarify diagnoses by revealing distinct metabolic patterns, making it a crucial diagnostic biomarker (2). In head-to-head studies, FDG-PET stereotactic surface projection (SSP) metabolic and statistical maps have been superior to clinical assessment regarding interrater reliability and diagnostic accuracy (3). It is a valuable complement to other diagnostic tools, as it enhances staging and monitoring of the extent and location of neurodegenerative disease, particularly tracking the progression of the disease over time (2, 4). In turn, earlier diagnosis with FDG-PET and confirmation with amyloid-PET and tau-PET can assist physicians in enrolling patients with clinical trials and pathology-specific disease-modifying therapies (5–7).

FDG-PET works by administering a radioactive tracer, [^18^F]-FDG, which, when taken up by metabolically active glial and neural cells, emits positrons that are detected by a scanner and computed into an image highlighting regions of the brain with increased or decreased glucose uptake (8). Diagnosing neurodegenerative diseases based on patterns of decreased glucose uptake was previously not possible using imaging techniques such as computed tomography (CT) or MRI, which mainly focused on detecting cortical atrophy (5). AD, the most common cause of dementia, is typically characterized by decreased glucose metabolism in the precuneus, posterior cingulate gyrus, and temporoparietal cortex, with sparing of the anterior cingulate, perirolandic region, visual cortex, basal ganglia, thalami, occipital lobes, brainstem and cerebellum (9, 10). FTD demonstrates a pattern of decreased glucose uptake in the frontal and anterior temporal lobes, with common involvement of the caudate nuclei and orbitofrontal region (11, 12). DLB is distinguished by decreased glucose metabolism in the parieto-occipital and temporal regions, including in the medial occipital lobes (cuneus and primary visual cortex), with sparing of the posterior cingulate (cingulate island sign) (12–21).

FDG-PET is typically used with clinical data, neuropsychological assessment, additional imaging modalities such as MRI, and cCSF measurements of *β*-amyloid and tau to differentiate between neurodegenerative diseases (22, 23). FDG-PET evidence with an Alzheimer’s pattern may indicate a lumbar puncture to determine a CSF Amyloid Tau Index (ATI), where reduced CSF levels of Aβ42-decreased Aβ42/40 ratio and increased CSF levels of tau, and phosphorylated tau can be used to confirm the diagnosis of AD (24, 25). Similarly, an Amyloid PET that detects cerebral Aβ deposition would help confirm amyloid pathology (26–28). These tests provide pathological evidence of disease and are necessary for inclusion in clinical trials and to assign patients to novel antiamyloid treatments (29). Similarly, when FTD is suspected, FDG-PET can trigger further investigations, such as the utilization of other radiotracers, including tau PET (30, 31).

PET radiotracers, such as [18F]-florbetapir, [18F]-florbetaben, and [18F]-flutemetamol, can be used to localize cortical amyloid deposition, whereas [18F]-flortaucipir can be used to localize regions of the brain with tau accumulation (7, 32, 33). In most studies, FDG is used to detect hypometabolism. In less frequent cases, patients affected by autoimmune encephalopathy (AEI), which is a known cause of rapidly progressing dementia, may display variable increased or decreased FDG uptake (34–37).

FDG-PET imaging is best combined with structural imaging such as MRI (PET/MRI), which can provide information on lobar-specific atrophy patterns unique to different types of dementia and can be particularly useful in defining the contribution of vascular pathology to ongoing clinical and metabolic phenotypes, as well as detecting co-pathologies such as cerebral amyloid angiopathy (CAA) (23). Notably, MRI provides precise and consistent information, helping overcome anatomic localization difficulties inherent to PET. The ease of acquiring PET and MRI in a single session on a hybrid system minimizes patient discomfort while maximizing clinical information and optimizes the co-registration of both modalities (38).

While FDG-PET is beneficial in helping to ascertain the diagnosis of neurodegenerative diseases, physicians and researchers should be aware of its limitations, leading to artifactual findings, and consider a patient’s underlying comorbidities and medications. Diabetic medications, such as insulin and metformin, can affect FDG biodistribution into skeletal and cardiac tissue (39). It is generally recommended to discontinue metformin 48 h before FDG-PET to standardize scan quality, though some studies suggest modifying the protocol based on the specific scan requirements (40). Brown fat is a potential confounder during FDG-PET as it exhibits high FDG uptake (41). *β* Blockers, such as propranolol, have been used to reduce brown adipose uptake of FDG, which has been helpful in the evaluation of tumors (42). Medications that can alter the cerebral metabolism of FDG include sedatives, narcotics, antipsychotic medications, and steroids (43). Therefore, a thorough evaluation of the patient’s medications should be completed to ensure reproducibility.

Ultimately, FDG-PET’s utility in distinguishing between dementia subtypes can help guide treatment options, potentially optimizing the therapeutic approach, enabling the enrollment of patients in appropriate clinical trials (44, 45), and, importantly, informing prognosis. The psychosocial implications of improving diagnostic accuracy should also be emphasized: the emotional frustration associated with false or uncertain diagnoses can be challenging for patients and caregivers alike, hence the need to improve the sensitivity and specificity of diagnosis. Here, we present a single-institution case series (Northwell Health, New York) and review pertinent literature wherein FDG-PET was utilized to obtain an accurate diagnosis of neurodegenerative disease, thereby allowing proper management. Patients were seen by a neurologist and, where applicable, a psychiatrist, both specialists in neurodegenerative diseases. Imaging was performed in the appropriate neuroradiology and nuclear medicine departments and read by a board-certified, fellowship-trained neuroradiologist with over a decade of clinical and research expertise focused on dementia and neurodegenerative disorders.

## Methods

### Literature search strategy

A comprehensive literature search was completed following the Preferred Reporting Items for Systematic Reviews and Meta-Analyses (PRISMA) guidelines. Titles and abstracts in English were searched across the PubMed citation database. No date limit was used, and articles were updated until April 2023. The medical subjects’ headings (MeSH) terms were used: “FDG-PET” AND “dementia” OR “Alzheimer’s” OR “neurodegeneration” OR “frontotemporal dementia” OR “atypical parkinsonian syndrome” OR “primary progressive aphasia” OR “lewy body dementia.”

### Selection criteria

**Figure 1:**
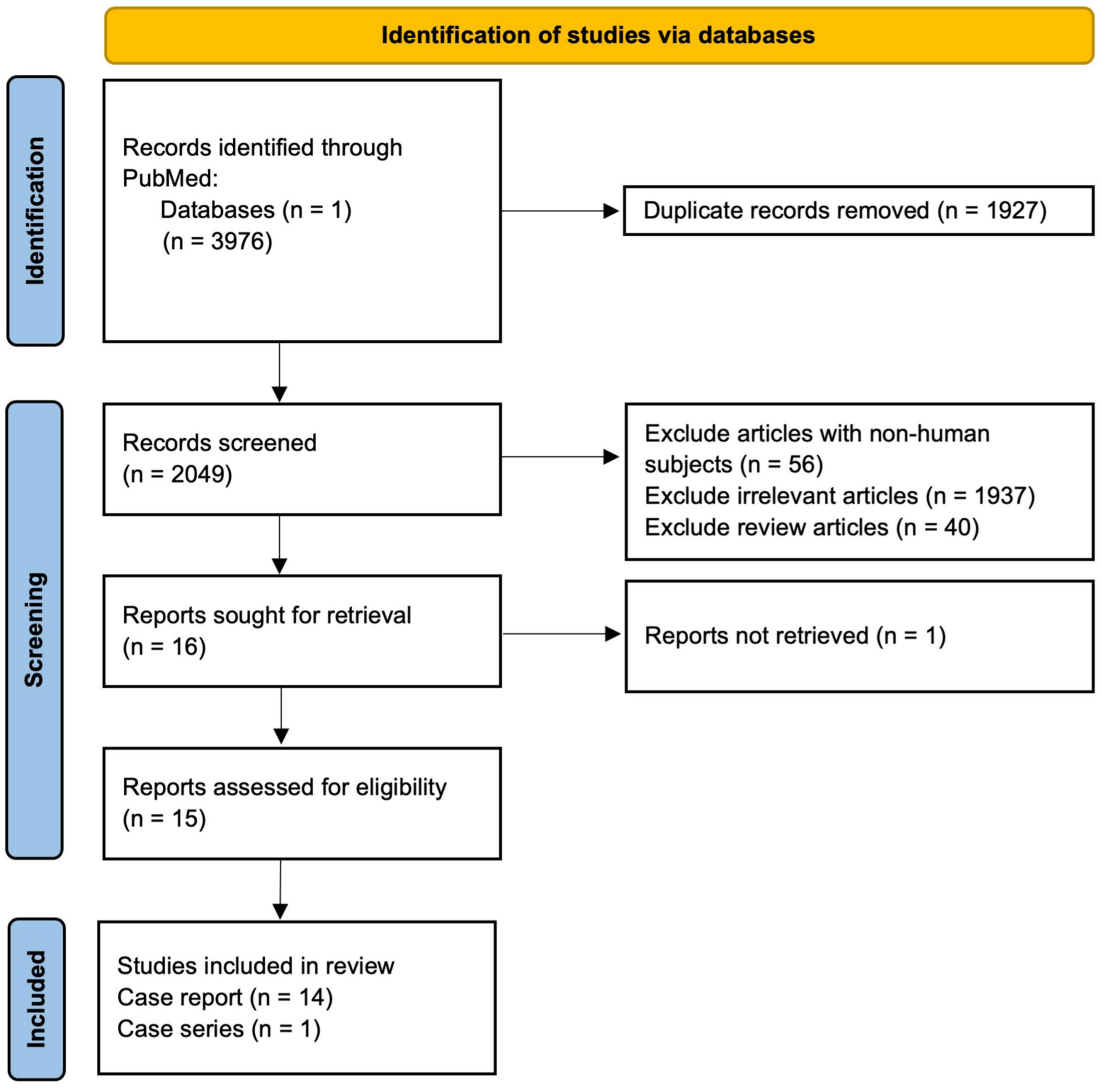
Preferred Reporting Items for Systematic Reviews and Meta-Analyses (PRISMA) schemata: diagrammatic representation of literature searches utilizing PubMed database.

The inclusion criteria were (1) studies with uncertain diagnoses of dementia that were resolved using FDG-PET, and (2) studies published in a peer-reviewed journal. The exclusion criteria consisted of studies with non-human subjects, reviews, editorials, expert opinion, abstract-only articles, articles unable to be obtained, and articles not relevant to the inclusion criteria. The search resulted in 3,976 results, of which 1,927 were removed for being duplicates. The remaining 2,049 articles were screened using the exclusion criteria, resulting in 16 relevant articles. One full-text article was unable to be obtained. In total, 15 reports were included, comprising 14 case reports and 1 case series of 5 patients (Figure 1; Table 1).

**Table 1:** Representative cases—systematic literature review.

Author	Number of patients	Principal neurological presentation	Original diagnosis	Final diagnosis	Age	Sex	Structural imaging	FDG-PET
Michels et al. (62)	1	Progressive cognitive impairment and mild orofacial dyskinesia MMSE: 24	Alzheimer’s disease	Huntington’s disease	54	M	No pathological findings	Bilateral hypometabolism in striatum
Obergassel et al. (63)	1	Acute loss of visual acuity, frontal–temporal headache, progressive olfactory dysfunction, but no cognitive impairment	Stroke	Creutzfeldt-Jakob disease	81	F	DWI signal in left occipital-parietal cortex with positive ADC correlate	Hypometabolism in the left occipital lobe and adjacent left parietotemporal and precuneus cortex areas
Pozzi et al. (54)	1	Cognitive impairment with frequent anomia MMSE improved from 16/30 to 28–29/30 after psychiatric treatment	AD	Pseudodementia	74	M	Mild diffuse cortical atrophy and age-related gliosis, stable over a 12-month period	Severe hypometabolism in the bilateral precuneus, temporal, and parietal lobes, which were resolved 12 months later after psychiatric treatment
Schönecker et al. (10)	1	Three-year history of progressive memory decline MMSE: 24	Cognitive decline of unclear etiology	AD	59	F	Normal at visual inspection; atlas-based volumetric analysis showed significant atrophy of temporal and parietal lobes (*Z*-scores for the hippocampal and parietal volumes −2.9 and − 4.2, respectively)	Asymmetric hypometabolism in parietal and temporal cortical areas and posterior cingulate, most severe on the right side
Suantio et al. (15)	1	Postural hypotension, recurrent visual hallucinations, Parkinsonism, and recurrent falls	Parkinson’s disease	DLB	72	M	Mild–moderate midbrain volume loss	Hypometabolism of bilateral occipital, posterior parietal lobe, and to a lesser extent, bilateral frontal lobes with relative sparing of the posterior cingulate gyrus and bilateral temporal lobes
Tun et al. (16)	1	Six years of gradual cognitive decline, visual hallucinations, acute period of altered consciousness, leading to a motor vehicle accident MMSE: 27	Epilepsy	DLB	83	M	No pathological findings	Hypometabolism in the bilateral occipital, parietal, and posterior temporal lobes
Van Der Gucht et al. (17)	1	Depression, delirium, recurrent visual hallucinations, and catatonic syndrome associated with cognitive decline	DLB vs. Alzheimer’s disease	DLB	67	M	Moderate cortical atrophy	Hypometabolism in the posterior associative cortex
Yoo et al. (18)	5	Four years of postural instability and stooping posture	Atypical Parkinsonian syndrome	DLB	76	M	N/A	Severe hypometabolism in bilateral occipital lobes
		Cognitive impairment, visual hallucination, dystonia, resting tremor, and bradykinesia	Atypical Parkinsonian syndrome	Corticobasal syndrome	73	M	N/A	Asymmetric hypometabolism in the right frontal, parietal, and temporal lobes
		Postural instability, akinesia, and cognitive impairment	Atypical Parkinsonian syndrome	Progressive supranuclear palsy	73	M	N/A	Moderate hypometabolism in bilateral frontal lobes
		Four years of urinary incontinence, orthostatic hypotension, parkinsonism symptoms, cerebellar signs such as gait and balance impairment and limb ataxia	Atypical Parkinsonian syndrome	Multiple system atrophy	78	M	N/A	Diffuse hypometabolism in the cerebellum
		Two years of bradykinesia, right upper extremity rigidity, and gait difficulty with recent falls	Atypical Parkinsonian syndrome	Multiple system atrophy	76	M	N/A	Hypometabolism in bilateral posterior putamen
Bouter et al. (19)	1	Ten years of recurrent depressive episodes, obsessive behavior, and progressive cognitive decline	DLB vs. Alzheimer’s disease	DLB	63	M	No pathological findings	Bilateral hypometabolism of frontal, occipital, parietal, and temporal lobe with relative sparing of the posterior cingulate gyrus
Caffarra et al. (55)	1	Twelve years of word-finding difficulties, executive dysfunction, and compulsive wandering and hoarding	Executive functions decline with paraphasia	Primary progressive aphasia	68	F	Atrophy in the left temporal and parietal lobes	Significant hypometabolism in the left hemisphere, angular and middle temporal gyri, precuneus, posterior cingulate, middle frontal gyrus, caudate, and bilaterally in the superior temporal and inferior frontal gyri
Di Battista et al. (11)	1	Depression, anxiety, and behavioral disturbances after Borreliosis infection MMSE:19	Lyme disease, major depressive disorder with psychosis	Frontotemporal dementia (FTD)	61	F	Frontal atrophy with moderate involvement of the anterior cingulate cortex and mild involvement of the frontal–insular and anterior temporal regions; widening of the posterior cingulate and parieto-occipital sulci; bilateral moderate–severe atrophy of the frontal motor cortices	Moderate-to-marked diffuse hypometabolism in all cerebral areas with severe involvement of the structures of the left lobe; mild hypometabolism in the left striatum
Gallucci et al. (12)	1	Six years of cognitive decline, sleep disturbances, and visual hallucinations	Parkinson’s disease	DLB and, later, behavioral-variant FTD with parkinsonism	79	M	Moderate-to-severe frontal cortical atrophy, more prominent on the right hemisphere, particularly, the mesial prefrontal cortex and anterior cingulate gyrus; subcortical atrophy with enlargement of lateral ventricles.	Hypometabolism of the frontal lobes and temporal lobes; reduced uptake in the right cerebellum
Gallucci et al. (20)	1	Depression, auditory hallucinations, gait impairment, and cognitive impairment	DLB	FTD	70	M	Moderate frontotemporal atrophy with no vascular lesions	1. Hypometabolism of the medial frontal cortex, parietal cortex, and occipital cortex; relative hypometabolism in the bilateral temporal region, precuneus, anterior cingulate cortex, posterior cingulate cortex, thalamus, and cerebellum in both hemispheres; 2. 7 months later, further hypometabolism was seen in the prefrontal and inferior temporal cortices, signifying the progression
Al-Faham et al. (21)	1	Three years of short-term memory loss with a decline in executive functioning, visuospatial functioning, memory, language, attention, and concentration MMSE: 6	DLB vs. AD	Dementia with Lewy bodies	71	M	N/A	Hypometabolism in left temporal, bilateral parietal, and occipital lobes
Borrelli et al. (64)	1	Five years of progressive cognitive decline characterized by impaired recall and recognition and severe aphasia with phonemic paraphasias	Lysosomal lipid storage disorder. e.g., adult-onset Niemann-Pick disease type C vs. multiple sclerosis	Logopenic variant of primary progressive aphasia (lvPPA) coexisting with multiple sclerosis	67	F	Bilateral non-enhancing periventricular, juxtacortical, and infratentorial demyelinating lesions	Hypometabolism in the left temporal and parietal lobes

AD, Alzheimer’s disease; DLB, dementia with Lewy bodies; FDG, fluorodeoxyglucose F18; FTD, frontotemporal dementia; PET, positron emission tomography; MMSE, mini-mental status examination.

## Case presentations

### Patient 1

A 67-year-old woman with baseline dementia, depression, and urinary incontinence presented for acute-on-chronic cognitive impairment. She was working as a high-functioning secretary until 2 years prior, after which cognitive issues prevented her from working further. She was noted to lose track of tasks and demonstrated difficulties with driving. The patient’s mood and sleep patterns had suffered during her illness. In the outpatient setting, the patient was empirically administered increasing doses of donepezil, with no improvement. She was diagnosed with mild cognitive impairment by her former neurologist and depression by her primary care physician.

**Figure 2:**
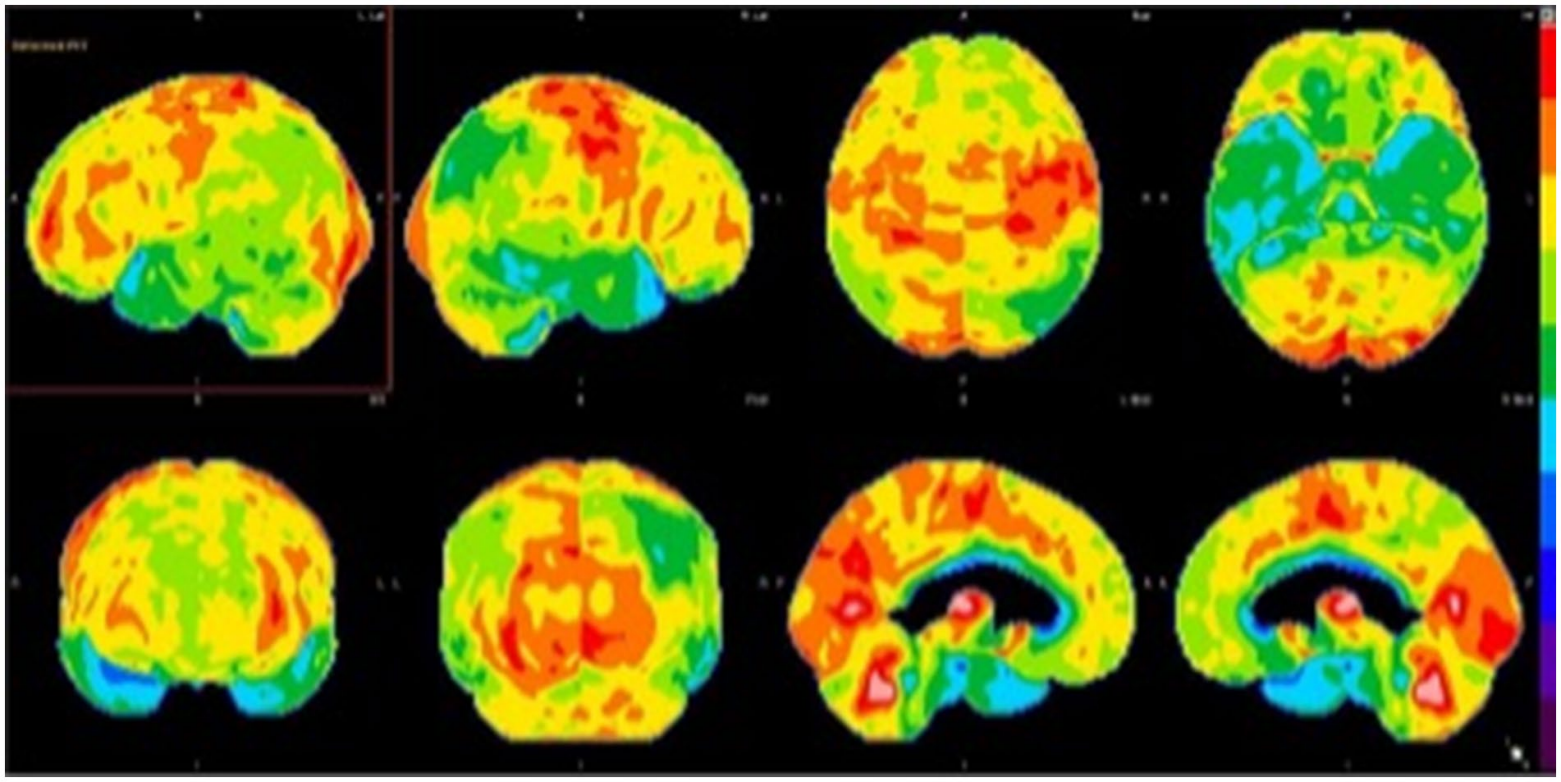
Brain FDG-PET imaging: 3D stereotactic surface projection maps demonstrate moderate hypometabolism in the parietotemporal regions (right greater than left), which extends into the frontal lobes. There is accompanying mild hypometabolism in the precuneus and posterior cingulate gyri, especially on the right side, regions typically involved early in the setting of Alzheimer’s disease.

When evaluated for a second opinion, she demonstrated deficits in clock drawing, trail-making, and remembering presidents. She was oriented to person, place, and time. She demonstrated neither signs of motor, dynamic, ideomotor, and ideational nor dressing apraxia. Her language ability was intact. She displayed adequate knowledge of personal and past history. She scored 24/30 on her mini-mental status examination (MMSE). There were no recorded deficits in motor strength, coordination, or sensation. Serum studies demonstrated normal antinuclear antibodies (ANA), thiamine, anti-Ro, anti-La, thyroid stimulating hormone (TSH), free T3/free T4 (fT3/T4), thyroid peroxidase (TPO) and thyroglobulin (TG) antibodies, syphilis screen, borrelia IgG/IgM antibodies, C-reactive protein (CRP). Brain MRI showed mild atrophy in the cortical and subcortical structures in the temporal lobe but not immediately indicative of AD. Brain FDG-PET showed moderate hypometabolism in the parietotemporal regions (right greater than left), which extended into the frontal lobes. There was accompanying mild hypometabolism in the precuneus and posterior cingulate gyri, especially on the right side, regions typically involved early in the setting of AD (Figure 2). This pattern suggested moderately advanced AD. An amyloid PET supported a pathological diagnosis by demonstrating significant cortical *β*-amyloid deposition. The patient was successfully screened and enrolled in an AD clinical trial while her mood changes were addressed and managed pharmacologically and behaviorally.

### Patient 2

**Figure 3:**
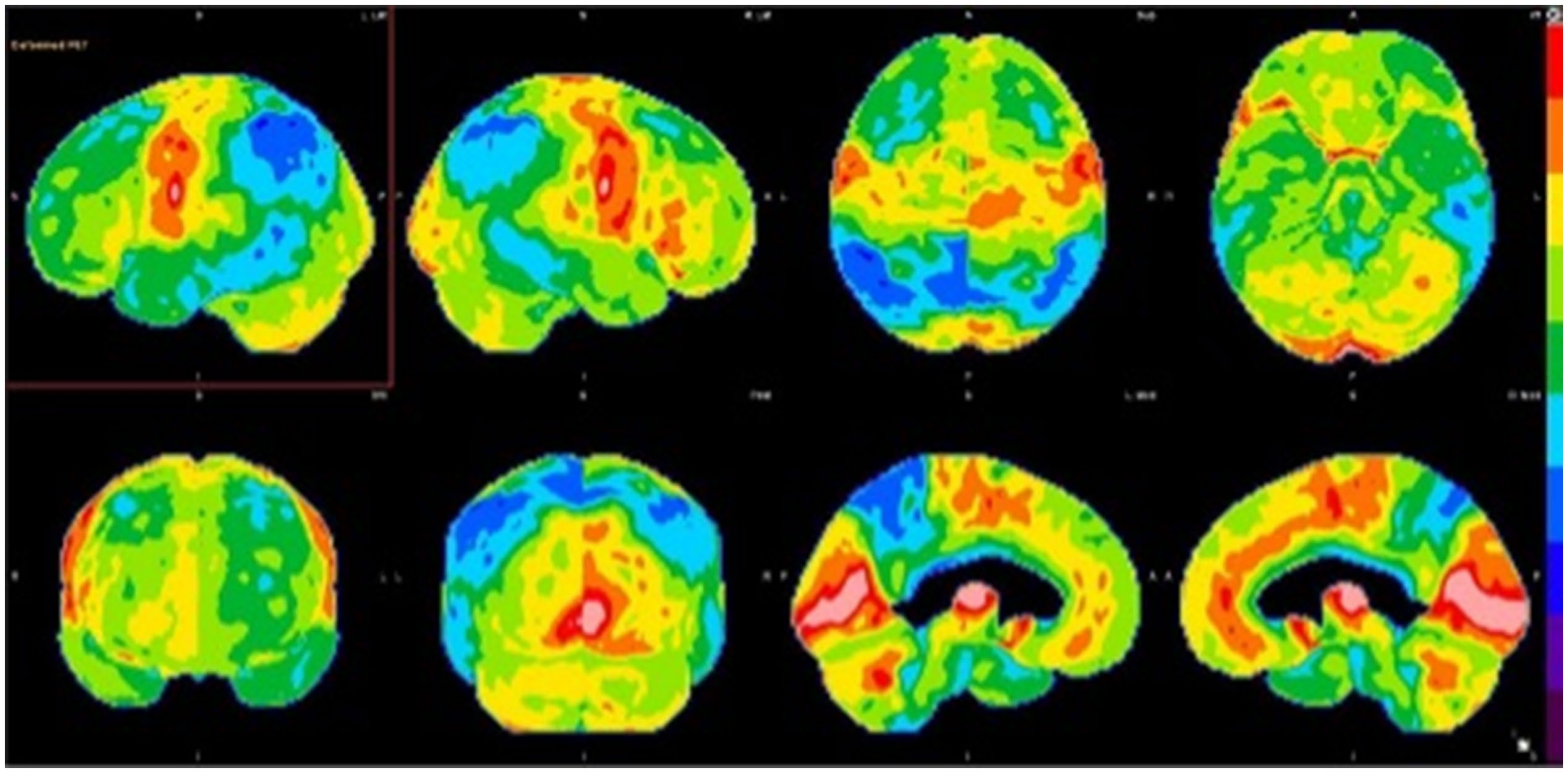
Brain FDG-PET imaging: 3D stereotactic surface projection maps demonstrate severe hypometabolism in the parietotemporal regions, left greater than right, with accompanying hypometabolism in the precuneus and posterior cingulate gyri bilaterally. There is extension of hypometabolism into the frontal lobes, indicating advanced neurodegenerative disease.

A 55-year-old man presented to the clinic with noted changes in behavior. He worked as an architect but lost his job due to cognitive decline. He complained of losing interest in many of his hobbies and forgetting basic tasks such as paying bills. On physical exam, the patient demonstrated full orientation, and difficulties with short-term memory and attention span but intact registration and concentration. There were neither language difficulties nor deficits in recalling current events. He scored 24/30 on his Montreal Cognitive Assessment (MoCA). Cranial nerve, motor, sensory, coordination, and reflexes were intact. Brain MRI demonstrated frontal atrophy and significant atrophy of the precuneus. Combined with apathy and depressed mood, the patient was diagnosed with a possible behavioral variant of frontotemporal dementia (bvFTD) and started on rivastigmine. Later, brain FDG-PET demonstrated severe hypometabolism in the parietotemporal regions, left greater than right, with accompanying hypometabolism in the precuneus and posterior cingulate gyri bilaterally (Figure 3). These findings were compatible with advanced AD rather than bvFTD. Subsequently, the patient was referred to and successfully enrolled in an AD clinical trial, with pathological confirmation supported by amyloid PET.

### Patient 3

A 60-year-old man with ongoing severe bipolar disorder presented for 1-year cognitive decline with acute exacerbation over the prior 2 months. Previously, he was a high-functioning individual but started to complain of difficulties following commands and staying organized with his thoughts.

**Figure 4:**
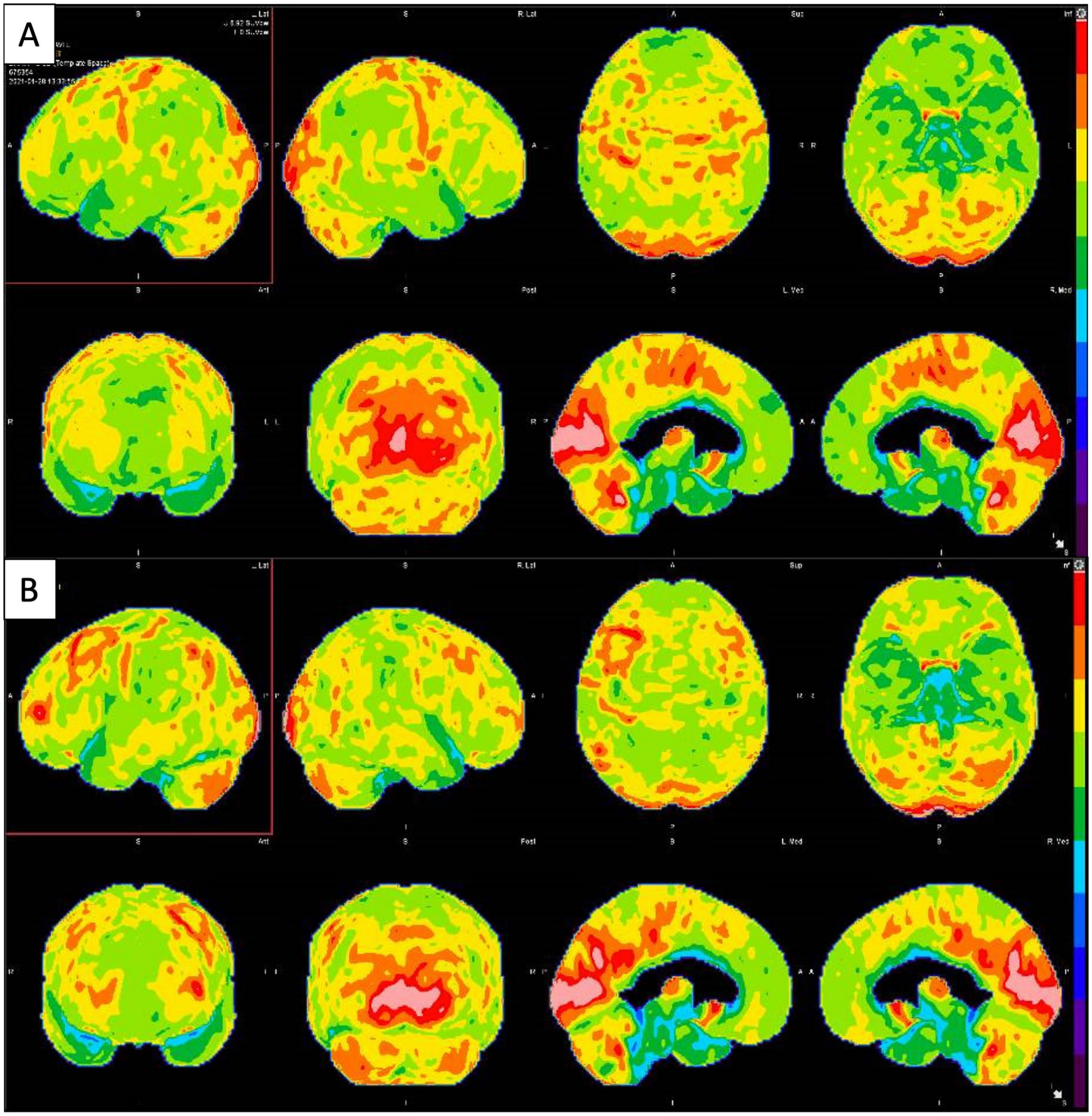
**(A)** Brain FDG-PET imaging: 3D stereotactic surface projection maps demonstrate mild to moderate hypometabolism in the bilateral parietotemporal regions (slightly more pronounced on the left side), which extends into the frontal lobes. There is accompanying mild hypometabolism in the precuneus and posterior cingulate gyri, regions typically involved early in the setting of Alzheimer’s disease. **(B)** Second Brain FDG-PET imaging taken 18 months after the first showing improvement in metabolic deficits.

On exam, the patient could recall four out of the previous five presidents. Upon neurocognitive testing, he was observed to have no difficulties with clock drawing, spiral drawing, and alternating patterns; he demonstrated noticeable errors in trail-making and fluency. During dynamic testing, testing for praxis was generally normal, with only difficulty in the left hand. He scored 26/30 on MMSE. Orientation, attention, language, fund of knowledge, cranial nerves, motor, coordination, and reflexes were normal or unimpaired. The sensory exam was generally intact with reduced vibration and position sense in the distal lower and upper extremities. Laboratory tests demonstrated negative encephalopathy antibody panel in serum (ENS1, Mayo Clinic Laboratories) and CSF (ENC1, Mayo Clinic Laboratories), syphilis screening, p-ANCA, c-ANCA, cryoglobulin, anti SS-A, SS-B, anti-Smith, erythrocyte sedimentation rate (ESR), antiribonucleoprotein and borrelia screening, and indeterminate atypical ANCA; normal levels of methylmalonic acid, homocysteine, vitamins B1, B2, B5, B6, B9, and B12, arsenic, lead, mercury, cadmium, TPO and TG antibodies, C-reactive protein, thyroid stimulating hormone (TSH), and fT3/T4. A brain MRI demonstrated no abnormal parenchymal or leptomeningeal enhancement and an incidental pituitary microadenoma. An FDG-PET, performed in the context of the acute changes, hospitalization, and significant medication regimen, demonstrated abnormal hypometabolism in the parietal–temporal regions with involvement of the precuneus and posterior cingulate gyri, extending into the frontal lobes (Figure 4A). Given the imaging findings, the patient was initially diagnosed with AD. The patient was started on donepezil with graduating doses. In the following months, the patient developed significant Parkinsonism and was noted to be significantly bradykinetic and rigid with asterixis. Upon further evaluation in our clinic for a second opinion, we conducted a full medication overhaul, with a reduction of anti-D2 and sedating medications, preferring targeted antidepressants and low-dose atypical antipsychotics, among others. He was followed for several months, and we noticed a significant improvement in his cognitive and motor status, while his behavioral issues remained persistent, to the point of being institutionalized for a brief period (also in relation to a lack of stable home and care by family). A repeated FDG-PET brain scan (18 months after the first FDG-PET) demonstrated interval improvement in metabolic deficits compared to the previous FDG-PET (Figure 4B). This confirmed the suspicion of pseudodementia, likely due to overmedication in the setting of bipolar disorder. Further psychiatric care is underway, with significant improvement in the patient’s behavioral status.

### Patient 4

An 82-year-old man with a previous diagnosis of pseudodementia due to depression presented with subacute onset of mild–moderate motor aphasia, initially ascribed to a possible stroke. Upon evaluation, brain MRI did not show significant vascular lesions. He was also noted to have unintentionally lost a significant amount of weight in relation to poor appetite and side effects of neurotropic medications; he was also in recent depressive episodes. His orientation, memory, attention, language, and fund of knowledge were intact in testing his mental status. He scored 27/30 on MMSE. He recalled 4/5 previous presidents. Testing for alternating patterns, spirals, and repetitions was normal. The clock drawing was abnormal as the patient drew numbers counterclockwise. He did not demonstrate apraxia though slow processing was noticeable.

**Figure 5:**
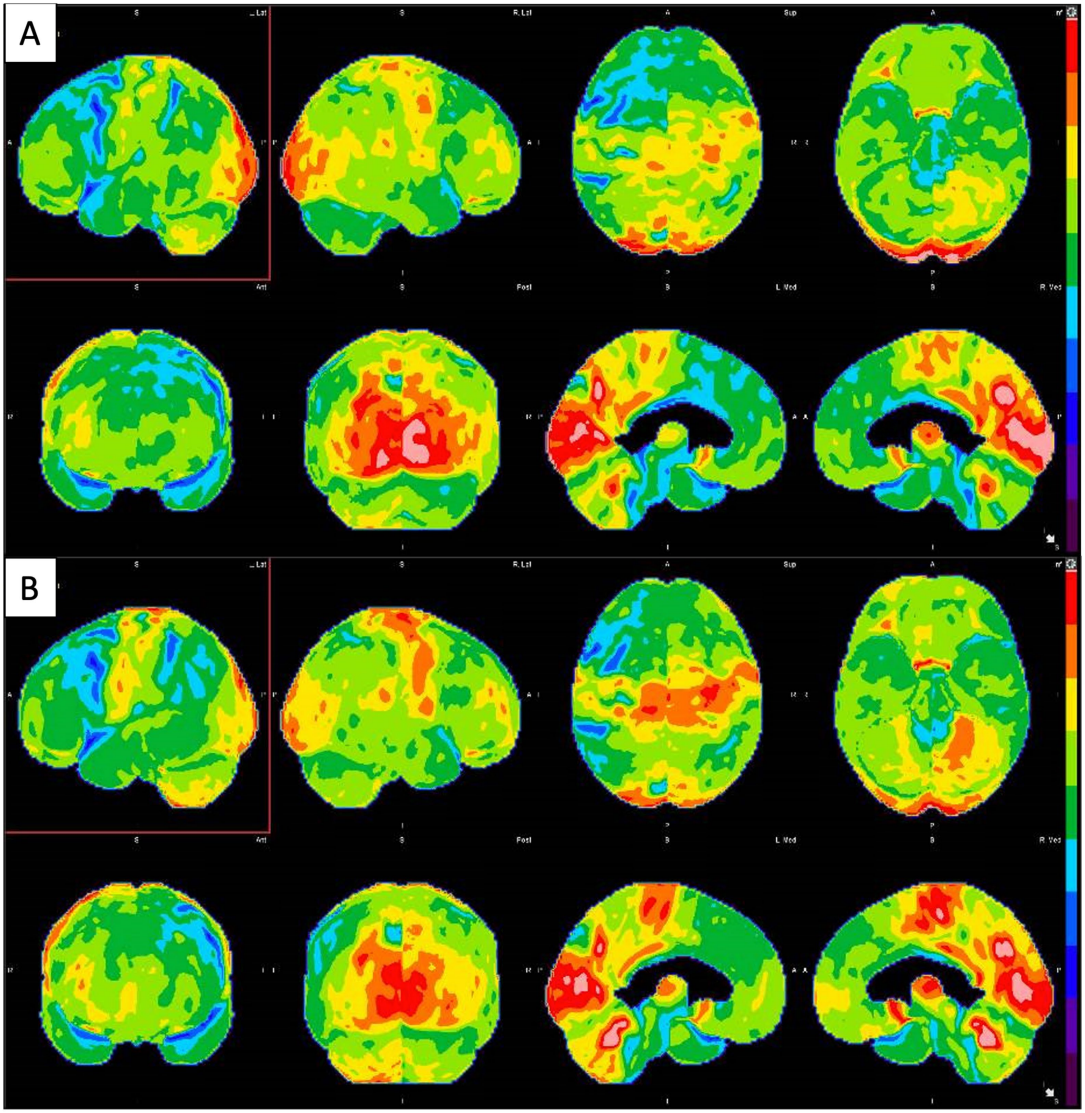
(A) Brain FDG-PET imaging: Three-dimensional stereotactic surface projection (SSP) maps demonstrate severe asymmetric hypometabolism in the left frontal lobe, particularly involving the left dorsolateral and fronto-insular region, including the left frontal operculum (pars opercularis and pars triangularis of the left inferior frontal gyrus, expected Broca’s area). There is accompanying crossed cerebellar diaschisis, with right greater than left cerebellar hypometabolism. (B) Second Brain FDG-PET imaging taken 12 months after the first showed similar hypometabolism distributions.

During neuropsychological testing, the patient understood the test and maintained adequate engagement. His thought processes were logical and goal directed. His speech was hesitant and notable for slow prosody and hypophonia. No spastic speech was elicited, but it was noticeable for being hesitant, halting with effort, and more limitation in gutturals. Brain FDG-PET demonstrated asymmetric hypometabolism in the left frontal lobe, including in the left dorsal frontal, peri-insular/perisylvian regions, with involvement of the left frontal operculum (pars triangularis and pars opercularis of the left inferior frontal gyrus—expected Broca’s area) and left supplementary motor area. (Figure 5A) Imaging also revealed right greater than left cerebellar hypometabolism, indicating a component of crossed cerebral diaschisis. Given neuropsychological testing and imaging, the patient’s condition was suggestive of corticobasal degeneration (CBD) with a non-fluent/agrammatic primary progressive aphasia (nfvPPA) component. The patient was started on several trials of cholinesterase inhibitors, which he did not tolerate; similarly, dopaminergic medications were started, with the benefit only from selegiline but also poorly tolerated. A year later, repeated PET-MRI demonstrated similar findings (Figure 5B). At this time, the patient’s clinical picture had progressed to CBD, with a profound nfvPPA phenotype.

## Results

Four patients were studied as part of our single-institution case series (Table 2). Nineteen patients were included as part of our literature review (Table 1). Six of these 23 patients were female. Two patients were diagnosed with AD before the age of 65 and, thus, qualified as early onset AD (10). Sixteen out of 23 patients had a brain MRI before FDG-PET for initial diagnosis. One patient had a CT scan (12). Six patients did not have an initial structural scan (18, 21). DLB was the most common neurodegenerative disorder that was initially misdiagnosed and then corrected through imaging (12, 15–19, 21). Most studies involved patients who were initially diagnosed with an alternative neurodegenerative disease and, through FDG-PET, were able to shift to the correct diagnosis. Some studies described patients with neurological symptoms, where the utilization of FDG-PET clarified the correct diagnosis (10, 17, 18).

**Table 2:** Single-institution case series.

	Principal neurological Presentation	Original diagnosis	Final diagnosis	Age	Sex	Structural imaging	FDG-PET
Patient 1	Two years of cognitive decline, depressive symptoms, and sleep disturbances MMSE: 24	Mild cognitive impairment vs. pseudodementia of depression	AD	67	F	Mild atrophy in the cortical and subcortical structures in the temporal lobe	Marked hypometabolism in left temporal lobe and bilateral (right greater than left) parietal lobes extending into right lateral occipital lobe; mild hypometabolism in bilateral frontal lobes and precuneus (right greater than left)
Patient 2	One year of cognitive decline, apathy, and hand tremors MMSE: 22	FTD	AD	55	M	Frontal lateral atrophy and significant atrophy of precuneus	1. Hypometabolism in bilateral parietal, parieto-occipital, and temporal regions, precuneus, and posterior cingulate gyrus; mild hypometabolism in the anteromedial frontal cortex bilaterally 2. 12 months later interval, improvement in metabolic deficits compared to previous PET imaging
Patient 3	One year of cognitive decline and parkinsonism symptoms MMSE: 26	AD	Pseudodementia	60	M	Small vessel white matter ischemic changes.	1. Hypometabolism in the parietal–temporal region, extending into the frontal lobes with involvement of the precuneus and posterior cingulate gyrus 2. 18 months later interval, improvement in metabolic deficits
Patient 4	Six years of cognitive decline, motor aphasia, parkinsonism symptoms, and fatigue MMSE: 27	Pseudodementia and vascular aphasia (stroke)	Corticobasal degeneration with nonfluent/agrammatic variant primary progressive aphasia	82	M	Confluent and patchy T2/FLAIR hyperintense changes in the periventricular subcortical white matter and in the central pons	1. Hypometabolism in the left dorsal frontal and peri-insular/peri-sylvian regions, the left frontal operculum and supplementary motor area, with accompanying volume loss on structural imaging; right greater than left cerebellar hypometabolism, indicating a component of crossed cerebral diaschisis 2. (12 months later)-similar hypometabolism in extent and distribution pattern

AD, Alzheimer’s disease; FDG, fluorodeoxyglucose F18; PET, positron emission tomography; MMSE, mini-mental status examination.

## Discussion

Brain FDG-PET is a desirable imaging modality that aids in the diagnosis of neurodegenerative disease. However, its cost may contribute to its limited use and availability, regardless of whether its radioisotopes are purchased or produced on-site (46). Our study highlights how FDG-PET can supplement or alter a preexisting clinical diagnosis. With an accurate diagnosis and in conjunction with pathological confirmation with amyloid and tau PET, patients may be started on treatment with resulting improvements seen through clinical symptoms and imaging (6, 15, 19, 47), or to access appropriate clinical trials (29, 48). FDG-PET should not be the sole study used to diagnose neurodegenerative disease. However, other imaging and biomarker studies are also fallible, especially when parsing through neurodegenerative diseases with similar initial presentations. FTD diagnosis can be challenging since its neurological symptoms can overlap with other neurodegenerative diseases, and its behavioral symptoms can mimic psychiatric disorders. Similarly, early presentation of DLB can be confused with those of AD, FTD, psychosis, and Parkinson’s disease dementia. The ability of FDG-PET to clarify some neurological diagnoses is becoming increasingly recognized, especially in the setting of emerging disease-modifying therapies that can lead to improved patient outcomes (27, 29, 48, 49). Of note, the combination of FDG-PET and clinical judgment can also lead to reversing the diagnosis of neurodegenerative dementia, as discussed in the subsection, “Patient 3.” FDG-PET diagnostic accuracy can be improved with several semi-quantitative methods. *Z*-scores measure a patient’s FDG update about the general population mean in terms of standard deviations and have been shown to improve the accuracy of readings (50). An abnormal finding would be reflected in a higher average *Z*-score (51). Centiloid scores are a semiquantitative method to improve accuracy in amyloid PET interpretation. The score quantifies the amyloid burden in the brain and normalizes the findings across different scanners and protocols, improving interstudy and intercenter interpretation (52, 53). The limitations of the current study lie in its retrospective nature. Thirteen patients in our study did not have an MMSE, which made it difficult to characterize the baseline cognitive impairment. In addition, only a few studies had a repeat MMSE following treatment (54). The patients described by Caffarra et al. (55) and Schönecker et al. (10) had clear symptoms of neurological decline but without a formal diagnosis. One patient underwent only computer tomography imaging but no MRI (12). The future studies can explore the use of other PET radiotracers, such as radiopharmaceuticals targeting tau (30), amyloid (32), FDOPA (56–58), TSPO (59, 60), and *α*-synuclein (61), in helping to diagnose neurodegenerative disorders accurately. In our current study, only a few patients were scanned with FDG and another tracer, such as amyloid PET (10, 17, 19). Others utilized single-photon emission computer tomography (SPECT) and dopamine active transporter (DaT) scans to supplement existing FDG-PET data (12, 17, 19, 20, 55).

In conclusion, FDG-PET can be a useful tool for diagnosing different subtypes of dementia with challenging clinical presentations. Additional investigation into the role of FDG-PET, for example, in psychiatric disorders, would be welcomed and may help improve the diagnosis and management of patients with dementia.

## Conclusion

The differential diagnosis of neurodegenerative disorders and dementia subtypes, including Alzheimer’s disease, FTD, dementia with Lewy bodies, atypical Parkinsonism, and pseudodementia can be challenging in patients with an unclear clinical presentation or inconclusive biomarkers. Dementia subtypes have unique FDG uptake patterns that can be used to aid in establishing an accurate diagnosis. Our case series and literature review highlight using FDG-PET brain imaging to correct and clarify preexisting clinical diagnoses of neurodegenerative disease.
